# Epidemiology and recent trends of severe sepsis in Spain: a nationwide population-based analysis (2006-2011)

**DOI:** 10.1186/s12879-014-0717-7

**Published:** 2014-12-21

**Authors:** Carmen Bouza, Teresa López-Cuadrado, Zuleika Saz-Parkinson, José María Amate-Blanco

**Affiliations:** Health-Care Technology Assessment Agency, National Center of Epidemiology, Instituto de Salud Carlos III, Madrid, Spain

**Keywords:** Severe sepsis, Epidemiology, General population, Health services research, Incidence, Mortality, Trends

## Abstract

**Background:**

Although severe sepsis constitutes an important burden for healthcare systems, there is limited nationwide data on its epidemiology in European countries. Our objective was to examine the most recent epidemiological characteristics and trends of severe sepsis in Spain, from a population perspective.

**Methods:**

Analysis of the 2006-2011 National Hospital Discharge Registry. Cases were identified by combining specific ICD-9CM codes. We estimated demographics, clinical characteristics and outcomes and calculated age- and sex- adjusted estimations of incidence and mortality rates. Trends were assessed in terms of annual percent change (APC) in rates using joinpoint regression analysis.

**Results:**

Over the 6-year period we identified 240939 cases of severe sepsis nationwide representing 1.1% of all hospitalisations and 54% of hospitalisations with sepsis. Incidence was 87 cases per 100,000 population. Overall 58% of cases were men, 66% were over the age of 65 and about 67% had associated comorbidities. Bacteremia was coded in 16% of records. Almost 54% of cases had one organ dysfunction, 26% two and around 20% three or more dysfunctions. In-hospital case-fatality was 43% and associated with age, gender, comorbidities and organ dysfunctions, among others. We found significant demographic and clinical changes over time with an increase in the mean age of cases, comorbidities, number of organ dysfunctions and in the number of cases with gram-negative pathogens. Furthermore, even with gender disparities, standardised incidence and mortality rates increased with an overall APC of 8.6% (95% CI 5.1, 12.1) and 6% (95% CI 1.9, 10.3), respectively. Conversely, we detect a significant decrease in case-fatality rates with an overall APC of -3.24% (95% CI: -4.2, -2.2).

**Conclusions:**

This nationwide population-based study shows that hospitalizations with severe sepsis are frequent and associated with substantial in-hospital mortality in Spain. Furthermore it indicates that the incidence and mortality rates of severe sepsis have notably increased in recent years, showing also a significant increase in the age and severity of the affected population. Despite this, there has been a significant decreasing trend in case-fatality rates over time. This information has significant implications for health-care system planning and may prove useful to estimate future care requirements.

**Electronic supplementary material:**

The online version of this article (doi:10.1186/s12879-014-0717-7) contains supplementary material, which is available to authorized users.

## Background

Severe sepsis is a substantial cause of morbidity and mortality worldwide and associates with high healthcare costs [1]-[4]. Recent estimates show that, in developed countries, severe sepsis is recorded in about 2% of patients admitted to hospital [1],[2], with case-fatality rates ranging from 30% to 50% [2],[4]. Furthermore, epidemiological studies in the USA have shown a progressive increase in its incidence, in excess of the growth of the population, driving an increase in the number of deaths [4]-[8]. Thus, severe sepsis constitutes both an important clinical and economic burden for healthcare systems and a major public health concern [1]-[5],[9],[10].

Despite this, there is limited information on the epidemiological characteristics of severe sepsis from a population perspective, particularly in Europe [11]-[13], and most research comes from intensive care units. However, in recent years it is more frequently being acknowledged that a large proportion of cases, between 50% and 70%, occur outside intensive care units [1],[2],[14],[15] limiting the extrapolation of results from studies carried out in this setting to the general population.

On the other hand, to date, most research providing population-based estimates [4]-[7] relies on hospital data samples that are weighted to extrapolate to national-level estimates and are, therefore, particularly vulnerable to sampling bias [16]. In this setting, national databases provide essential tools in epidemiological research of diseases such as severe sepsis where, on the one hand, it is impossible to prospectively identify patients at a nationwide level [1],[2],[4],[16],[17] and, on the other, it lacks the errors that are sometimes present in data obtained from reduced samples, specific groups, or collected in a short time period [16]. Consistent estimates of national incidence, mortality and other epidemiological data are critical steps for the assessment of the full burden of severe sepsis [4],[5],[18]. Besides, temporal data may facilitate the detection of trends in epidemiology [5].

Taking this into consideration, we undertook this study to examine the most recent epidemiological characteristics and trends of severe sepsis in Spain using the national registry of hospital discharges.

## Methods

### Data source

Information regarding hospitalized patients was collected from the National Minimum Basic Data Set (MBDS). The MBDS is the official database of the Ministry of Health, Social Services and Equality and collects demographic and clinical information on discharge of all acute-care hospital admissions nationwide. Information is gathered in the clinical documentation units of each hospital of the National HealthCare System by trained personnel and is merged, after a process of validation, into a single database. The information it provides is considered to be representative of the national population as it includes data on over 97% of all annual hospital admissions in our country [19].

National Population data come from the Spanish National Statistics Institute [20].

### Period of analysis

For study purposes we used the national database from January 1, 2006 to December 31, 2011.

### Selection of cases and definitions

Based on prior studies [4]-[7],[18], severe sepsis cases were identified as the presence, in principal or secondary diagnoses, of ICD-9CM (International Classification of Diseases, 9th revision, Clinical Modification) codes for sepsis and acute organ dysfunction or the presence of ICD-9CM code 995.92 specific of severe sepsis (systemic inflammatory response syndrome due to infectious process with organ dysfunction).

To identify sepsis we employed formerly utilized codes [5]-[7] that define infection: 038 (0.38.0 (streptococcal septicemia), 038.1 (staphylococcal septicemia), 038.2 (pneumococcal septicemia), 038.3 (septicemia due to anaerobes), 038.4 (septicemia due to other Gram negative organisms), 038.8 (other specified septicemias), 038.9 (unspecified septicemia), 003.1 (salmonella septicemia); 020.2 (septicemic plague); 036.2 (meningococcal septicemia); 036.3 (Waterhouse-Friderichsen syndrome); 054.5 (herpetic septicemia); 098.89 (gonococcemia); 112.5 (systemic candidiasis); 112.81 (candidal endocarditis); 117.9 (other and unspecified mycoses); 771.8 ( perinatal infections, septicaemia of newborn ) and 790.7 (bacteremia). We also included the ICD-9CM code for sepsis 995.91 (sepsis, systemic inflammatory response syndrome due to infectious process without organ dysfunction), that became effective in our country in January 2004 [19].

For acute organ dysfunction we used the following ICD-9CM codes [5],[6],[14]: respiratory: 518.81 (acute respiratory failure), 518.82 (other pulmonary insufficiency), 518.84 (acute on chronic respiratory failure), 518.85 (acute respiratory distress syndrome after shock or trauma), 786.09 (respiratory distress, insufficiency), 799.1 (respiratory arrest), 96.7(invasive mechanical ventilation); cardiovascular: 785.5 with all subcodes (shock without mention of trauma, includes 785.1, 785.52, 785.9),458 (hypotension, 458.0, 458.8 458.9), 796.3 (nonspecific low blood pressure reading); renal: 584 with all subcodes (acute renal failure), 580 (acute glomerulonephritis), 39.95 (hemodialysis); hepatic: 570 (acute and subacute necrosis of liver), 572.2 (hepatic coma), 573.3 (hepatitis, unspecified); hematologic: 286.6 (defibrination syndrome), 286.9 (other and unspecified coagulation defects), 287.3-5 (secondary thrombocytopenia, unspecified); neurologic: 293 (acute delirium), 348.1 (anoxic brain damage), 348.3 (encephalopathy, unspecified), 357.82 (critical illness polyneuropathy), 780.01 (coma), 780.09 (drowsiness, unconsciousness, stupor), 89.14 (electroencephalogram) and metabolic: 276.2 (acidosis metabolic or lactic).

To explore the extent of comorbidities of known prognostic value we used a validated ICD-9CM version of the Charlson Index [21]. Prior population-based epidemiological studies in sepsis have shown that there is no overlap between the codes used to calculate this index and the diagnostic codes used for acute organ dysfunction [14]. For this study, four different score groups (0, 1-2, 3-4 and > 4) were considered [22].

We defined cases as surgical if they had a major surgical procedure other than tracheostomy [4] based on the Diagnosis-related group codes [19].

### Ethical aspects

The study was exempt from institutional review board approval because only de-identified data were used [23].

### Data analysis

Initially we conducted a descriptive study to ascertain demographic characteristics, underlying comorbidities, Charlson index scores, number and type of organ dysfunctions and case-fatality. Data are summarised as frequency tables and percentages for categorical variables and summary statistics (mean ± SE) for continuous variables. For between-group comparisons, we used the Snedecor’s F for continuous variables and the χ2 tests for categorical data. We performed an exploratory logistic regression analysis to identify factors associated with in-hospital mortality. Factors that had strong biological relevance, such as gender, age-group and Charlson index, or were significantly related to hospital outcomes, such as the number of organ failures, were included in the model. Odds ratios (ORs) with 95% confidence intervals were computed.

Incidence and mortality rates were estimated using national population data expressing the results per 100000 population. Case-fatality rates (CFR) were calculated as the number of deaths divided by the number of cases, expressed as a percentage [6],[7]. Age and sex-adjusted rates were calculated by direct methods based on the European standard population [24]. Age and sex-adjusted CFR was calculated by direct standardisation based on the 2008 data.

To identify trends in incidence, mortality and case-fatality rates we quantified the annual percentage change (APC) with its respective 95% confidence intervals using the Joinpoint Regression Program [25]. APC is a widely used parameter to evaluate trends [26], and we used linear-log regression models assuming a Poisson distribution for its estimation [27]. This procedure enables testing whether an apparent change in trend is statistically significant using a Monte Carlo permutation method [27].

Statistical analysis was performed using STATA 12 (© 1996–2012 StataCorp LP. TX 77845 USA). P-values < 0.05 were considered significant.

## Results

Out of the 22,070,672 hospital discharges that occurred in Spain from 2006 to 2011, and after several processes of verification and depuration of the database, we identified 240939 overnight hospitalizations with severe sepsis.

### General characteristics

Descriptive characteristics of the population are shown in Table 1. Mean age was 65 years, 66% of episodes occurred in people ≥ 65 years of age and 58% of cases were male. Mean Charlson Index score was 1.9. ± 0.004. Around 67% of cases had underlying comorbidities ranging from 12% in those younger than 18 years to 65% in 18-64 year olds and 75% in those ≥ 65 years of age. Non-metastatic cancer, chronic obstructive pulmonary disease, chronic heart failure and chronic renal disorders were, overall, the most common categories of comorbidity.

**Table 1:** **General characteristics of the population (n = 240939)**

Characteristic	Number of cases, (%)
**Sex**	
Men	140202 (58.2)
Women	100728 (41.8)
**Mean age (yr)**	65.5 ± 0.05
**Age-group**	
<18 y	18093 (7.5)
18-64 y	64764 (26.9)
>64 y	158082 (65.6)
**Charlson index**	
0 points	78665 (32.7)
1-2 points	100530 (41.7)
3-4 points	37901 (15.7)
>4 points	23843 (9.9)
**Comorbidities** ^**a**^	
Cancer (non-metastatic)	41180 (17.1)
Chronic heart failure	34293 (14.2)
Chronic Obstructive Pulmonary Disease	32191 (13.4)
Chronic renal disease	32206 (13.4)
Cerebrovascular disease	20033 (8.3)
Cancer (metastatic)	14313 (5.9)
Acute myocardial infarction	9412 (3.9
Chronic liver disease	8449 (3.5)
Diabetes with complications	6179 (2.6)
Peripheral vascular disease	5143 (2.2)
AIDS	2936 (2.1)
**Diagnostic categories**	
Medical	178619 (74.1)
Surgical	62320 (25.9)
**Identified pathogens** ^**a**^	
**Yes**	61944 (25.7)
Gram-positive	22889 (37)
Gram-negative	37162 (60)
Anaerobic	988 (1.6)
Fungus	4566 (7.4)
**No. of organ system dysfunctions**	
1	128940 (53.5)
2	62874 (26.1)
≥3	49125 (20.4)
**Organ system dysfunction** ^**a**^	
Respiratory	121496 (50.5)
Cardiovascular	107872 (44.7)
Renal	97699 (40.5)
Hematologic	28382 (11.8)
Hepatic	12586 (5.2)
Metabolic	20049 (8.3)
Neurologic	26449 (11)
**Mean length of hospital stay (days)**	23 ± 0.06
**In-hospital deaths**	104461 (43)

Data presented as number of cases and %. ^a^Proportions not mutually exclusive.

Medical diagnostic categories were majority with surgical diagnoses identified in only 26% of cases. A microbiological diagnosis of the infection was registered in 61,944 (25.7%) cases. The frequency of the identified pathogens appears in Table 1, with gram-negative bacteria being the most common. Bacteraemia was coded in 39,810 cases.

Around 54% of cases had one organ dysfunction, 26% experienced two and about 20% three or more dysfunctions. The most frequent dysfunctions were respiratory (51% of cases), cardiovascular (45%) and renal (40%).

There were 103,461 deaths, which corresponds to a crude in-hospital case-fatality of 43%. The mean age of non-survivors was 71.4 ± 0.06 years and the mean Charlson Index score was 2.2 ± 0.007. As Table 2 shows, mortality varied with several demographic and clinical characteristics. Bivariate and multivariate analyses are also shown in Table 2. Adjusted multivariate logistic regression analysis reveals that the risk of death was significantly associated with age, severity of comorbidities, the presence of a subjacent medical diagnostic category, absence of identified pathogens, and with an increasing number of failing organs. It also shows that men have a lower risk of death than women. In addition, homogeneity test provide evidence that age acts as an effect modifier of all other covariates (data not shown).

**Table 2:** **In-hospital deaths**

Characteristic	Cases (%)	Case-fatality (% severe sepsis)	Bivariate OR (CI 95%)	Multivariate OR (CI 95%)
**Sex**				
Men	59424 (57)	42.4	Reference group	Reference group
Women	44037(43)	43.7	1.06 (1.04, 1.07)	1.12 (1.10, 1.14)
**Age-group**				
<18 y	3211 (3)	17.8	Reference group	Reference group
18-64 y	22588 (22)	34.9	2.48 (2.38, 2.59)	1.56 (1.49, 1.63)
>64 y	77665 (75)	49.1	4.48 (4.30, 4.66)	3.20 (3.06, 3.33)
**Charlson index**				
0	26540 (26)	33.7	Reference group	Reference group
1-2	45287 (44)	45.1	1.61 (1.58, 1.64)	1.34 (1.31, 1.37)
3-4	18331 (18)	48.4	1.84 (1.79, 1.89)	1.57 (1.52, 1.61)
>4	13306 (13)	55.8	2.48 (2.41, 2.55)	2.58 (2.50, 2.66)
**Diagnostic categories**				
Surgical	27655 (26.7)	44.4	Reference group	Reference group
Medical	75809 (73.3)	42.4	0.93 (0.91, 0.94)	1.03 (1.01, 1.06)
**Identified pathogens**				
No	40151 (78.9)	45.6	Reference group	Reference group
Yes	21793 (21.1)	35.2	0.65 (0.63, 0.66)	0.63 (0.62, 0.65)
**No. of organ system dysfunctions**				
1	43023 (41.6)	33.4	Reference group	Reference group
2	29712 (28.7)	47.3	1.78 (1.75, 1.82)	1.88 (1.84, 1.92)
≥3	30729 (29.7)	62.6	3.34 (3.26, 3.41)	3.89 (3.80, 3.98)

General characteristics, case-fatality and risk.

OR, odds ratio; CI 95%, 95% confidence interval.

As shown in Table 3, within the study period there have been several demographic and clinical changes. From 2006 to 2011 the mean age of cases increased significantly both in men and women related to a clear increase in the number of cases over the age of 65 years. Likewise, a significant increase in the Charlson index score is observed, as well as an increase in some specific comorbidities and, in particular, an increase in cases with chronic heart and renal disorders. Specifically, the percentage of cases with chronic renal disease doubled during the period of study.

**Table 3:** **Characteristics of the episodes of severe sepsis: Sequential data from 2006 to 2011**

	2006	2007	2008	2009	2010	2011
**Men**	59.2% (58.7, 59.8)	58.8% (58.3, 59.3)	58.4% (57.9, 58.9)	58.4% (57.9, 58.8)	57.8%(57.3, 58.3)	57.2% (56.8, 57.7)
**Age**	62.7 ± 0.15	63.9 ± 0.13	64.6 ± 0.12	65.7 ± 0.11	67.0 ± 0.11	67.6 ± 0.10
Men	61.2 ± 0.19	62.6 ± 0.16	63.2 ± 0.16	64.5 ± 0.14	65.9 ± 0.14	66.1 ± 0.13
Women	64.8 ± 0.23	65.7 ± 0.19	66.7 ± 0.17	67.5 ± 0.16	68.5 ± 0.17	69.5 ± 0.16
**Age groups**						
<18 y	9.7% (9.4, 10.0)	8.6% (8.3, 8.9)	8.1% (7.9, 8.4)	7.3% (7.1, 7.5)	6.6% (6.3, 6.8)	6% (5.8, 6.3)
18-64 y	28.5% (28.0, 29.0)	28.1% (27.6, 28.6)	27.7% (27.3, 28.1)	26.8% (26.4, 27.2)	25.6% (25.2, 26.0)	25.7% (25.3, 26.0)
>64 y	61.8% (61.2, 62.4)	63.3% (62.8, 63.8)	64.2% (63.7, 64.6)	65.9% (65.5, 66.3)	67.8% (67.4, 68.2)	68.3% (67.9, 68.7)
**Diagnostic categories**						
Medical	75.3% (74.8, 75.8)	75.1%(74.7, 75.6)	72.8%(72.3, 73.2)	73.1% (72.7, 73.5)	74.1% (73.7, 74.5)	74.8% (74.4, 75.2)
Surgical	24.7% (24.2, 25.2)	24.9%(24.4, 25.3)	27.2% (26.8, 27.7)	26.9% (26.5, 27.3)	25.9% (25.5, 26.3)	25.2% (24.8, 25.6)
**Charlson index**	1.76 ± 0.01	1.73 ± 0.01	1.84 ± 0.01	1.90 ± 0.01	1.91 ± 0.01	1.95 ± 0.01
**Comorbidities**						
Cancer (non-metastatic)	17.0% (16.6, 17.4)	16.8%(16.4, 17.2)	16.7% (16.3, 17.1)	17% (16.6, 17.4)	17.2% (16.8, 17.5)	17.7% (17.3, 18.0)
COPD^a^	12.9% (12.5, 13.3)	13.2%(12.8, 13.6)	13.4% (13.1, 13.7)	13.9% (13.6, 14.2)	13.3% (13.0, 13.7)	13.2% (12.9, 13.5)
Chronic cardiac failure	12.5% (12.1, 12.9)	13.1% (12.8, 13.5)	13.9% (13.5, 14.2)	14.6% (14.3, 14.9)	14.9% (14.5, 15.2)	15.4% (15.1, 15.7)
Chronic renal disease	8.8% (8.5, 9.2)	8.8% (8.5, 9.1)	13.3% (13.0, 13.6)	14.9% (14.5, 15.2)	15.4% (15.0, 15.7)	16.1% (15.8, 16.4)
Cerebrovascular	8.4% (8.1, 8.8)	8.1% (7.8, 8.4)	8.3% (8.0, 8.5)	8.4% (8.0, 8.5)	8.4% (8.1, 8.6)	8.3% (8.1, 8.6)
Cancer with metastasis	5.6% (5.3, 5.9)	5.5% (5.3, 5.7)	5.7% (5.4, 5.9)	5.8% (5.6, 6.1)	6.3% (6.1, 6.5)	6.4% (6.2, 6.6)
**Number of organ dysfunctions**						
1	55.1% (54.5, 55.6)	54.1% (53.6, 54.7)	53.9% (53.4, 54.3)	53.1% (52.6, 53.5)	53.9% (53.4, 54.3)	52.0% (51.6, 52.4)
2	26.8% (26.3, 27.3)	26.2% (25.7, 26.7)	25.9% (25.5, 26.3)	26.1% (25.6, 26.5)	25.5% (25.1. 25.9)	26.4% (26.0, 26.8)
≥3	18.2% (17.7, 18.6)	19.7% (19.3, 20.1)	20.3% (19.9, 20.6)	20.9% (20.5, 21.3)	20.7% (20.3, 21.0)	21.6% (21.3, 22.0)
**Type of organ dysfunction** ^**b**^						
Respiratory	51.9% (51.4, 52.5)	52.3% (51.8, 52.8)	51.6% (51.1, 52.1)	51.6% (51.1, 52.0)	48.9% (48.4, 49.4)	47.7% (47.3, 48.1)
Cardiovascular	45.9% (45.3, 46.4)	47.0% (46.5, 47.5)	45.9% (45.4, 46.4)	44.4% (44.0, 44.9)	43.9% (43.4, 44.3)	42.8% (42.3, 43.2)
Renal	36.5% (36.0, 37.1)	37.0% (36.5, 37.6)	38.2% (37.7, 38.6)	40.1% (39.6, 40.5)	41.7% (41.3, 42.2)	46.5% (46.1, 47.0)
Hepatic	5.3% (5.1, 5.6)	5.2% (5.0, 5.5)	5.4% (5.2, 5.7)	5.4% (5.2, 5.7)	5.1% (4.9, 5.3)	4.9% (4.7, 5.1)
Metabolic	7.4% (7.1, 7.7)	7.2% (7.0, 7.5)	7.8% (7.5, 8.0)	8.7% (8.4, 8.9)	8.7% (8.5, 9.0)	9.4% (9.1, 9.6)
Neurologic	10.6% (10.3, 11.0)	10.6% (10.3, 11.0)	10.8% (10.5, 11.1)	11.2% (10.9, 11.5)	11.1% (10.8, 11.4)	11.2% (11.0, 11.5)
Hematolologic	11.1% (10.7, 11.5)	11.0% (10.7, 11.4)	11.8% (11.5, 12.1)	11.8% (11.5, 12.1)	11.9% (11.6, 12.2)	12.4% (12.1, 12.7)
**Identified Germs** ^**b**^						
**Yes**	26.9% (26.4, 27.4)	25.6% (25.1, 26.0)	25.4% (25.0, 25.8)	24.8% (24.4, 25.2)	25.8% (25.4, 26.2)	26.1% (25.8, 26.5)
Gram positive	39.4% (38.4, 40.5)	39.2% (38.2, 40.3)	39.1% (38.2, 40.1)	37.3% (36.4, 38.3)	35.1% (34.3, 36.0)	33.6% (32.8, 34.4)
Gram-negative	56.3% (55.2, 57.4)	56.3% (55.2, 57.3)	58.1% (57.1, 59.0)	59.2% (58.3, 60.1)	62.4% (61.5, 63.3)	64.5% (63.7, 65.4)
Anaerobic	1.5% (1.2, 1.8)	1.5% (1.3, 1.8)	1.4% (1.2, 1.7)	1.7% (1.5, 2.0)	1.5% (1.3, 1.8)	1.8% (1.6, 2.0)
Fungus	9.4% (8.7, 10.0)	8.7% (8.1, 9.3)	7.4% (6.9, 7.9)	7% (6.5, 7.5)	6.8% (6.3, 7.2)	6.1% (5.8, 6.6)
**Bacteremia**	18% (17.6, 18.5)	16.4% (16.0, 16.8)	16.2% (15.9, 16.6)	16.4% (16.0, 16.7)	16.2% (15.9, 16.6)	16.4% (16.0, 16.7)
**Length of stay (days)**	24.8 ± 0.19	24.5 ± 0.17	24.1 ± 0.16	23.1 ± 0.14	21.9 ± 0.13	21.0 ± 0.12
**Case-fatality**	45.4% (44.9, 46.0)	45.9% (45.4, 46.5)	43.6% (43.1, 44.1)	42.9% (42.5, 43.4)	41.5% (41.0, 41.9)	40.2% (39.8. 40.6)

^a^Chronic Obstructive Pulmonary Disease. ^b^Proportions not mutually exclusive.

Data presented as % of cases (with its corresponding 95% Confidence Interval) or mean ± SE.

There are also significant changes with time in the number as well as type of organ dysfunctions. Thus, a small decrease in the percentage of cases having a single dysfunction is seen (55% in 2006, 52% in 2011), while there is an increase in cases with three or more dysfunctions (18% in 2006, 22% in 2011). Additionally, there are significant changes in the type of dysfunctions being relevant the increasing frequency of renal failure between 2006 (36%) and 2011 (46% of cases). As regards to microbiological data, the most striking finding seems to be the decreasing trend of gram-positive pathogens and the increase of records with gram-negative bacteria.

As Table 3 shows, within the study period hospital case-fatality decreased significantly from 45% of cases in 2006 to 40% in 2011. Data also show a relevant decrease in the length of hospital stay over time.

### Incidence, mortality and case-fatality rates

These cases represent 1.1% of national hospitalisations and 54% of hospitalisations with sepsis from 2006 to 2011. The overall incidence of severe sepsis was 86.97 cases per 100,000 population. In men, the crude incidence rate was 102.47 cases per 100000 population whereas in women it was of 71.90 cases per 100000 population.

**Figure 1:**
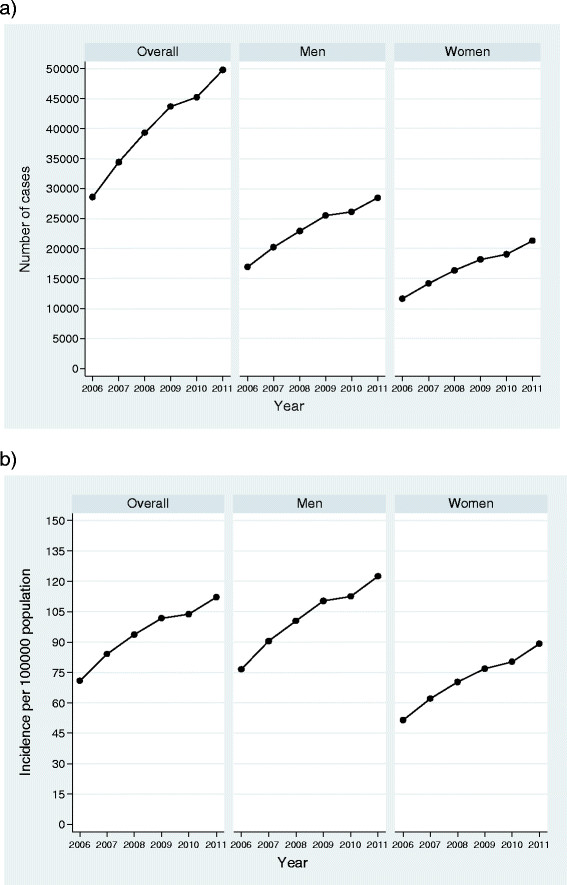
**Number of cases and age-adjusted national incidence of severe sepsis in Spain from 2006 to 2011.** The data correspond to the annual number of cases (plot 1**a**) and the age-adjusted standardized incidence rates (plot 1**b**) for the whole population of severe sepsis and for men and women in a specific manner. Rates were calculated by direct methods based on the European standard population. For the entire population, cases raised from 28579 in 2006 to 49782 in 2011. The standardised rate increased from 70.86 in 2006 to 112.11 cases/100000 population in 2011. In men (central section), cases have gone from 16927 in 2006 to 28488 in 2011. The standardised rate has increased from 95.02 cases/100000 population in 2006 to 147.05 cases/100000 population in 2011. In women, cases have gone from 11651 to 21293 and the standardised incidence rate has increased from 51.73 cases/100000 population in 2006 to 84.57 cases/100000 population in 2011.

There have been significant annual changes in incidence over time increasing from 63.91 cases/100000 population in 2006 to 105.51 cases/100000 population in 2011. The standardised rate increased from 70.86 cases/100000 population in 2006 to 112.11 cases/100000 population in 2011, with an APC of 8.6% (95% CI: 5.1, 12.1) (Figure 1).

Figure 1 also shows trends for men and women. In men, the crude rate has gone from 76.58 cases/100000 population in 2006 to 122.35 cases/100000 population in 2011. The standardised rate has increased from 95.02 cases/100000 population in 2006 to 147.05 cases/100,000 population in 2011, with an APC of 8.2% (95% CI: 4.7, 11.8).

For women, the crude incidence rate has gone from 51.52 cases/100000 population in 2006 to 89.12 cases/100000 population in 2011. The standardised rate has increased from 51.73 cases/100000 population in 2006 to 84.57 cases/100000 population in 2011, with an APC of 9.20% (95% CI: 5.8, 12.7).

The overall mortality rate during the study period was 37.1 cases per 100000 population. In men, the crude mortality rate was 43.15 cases per 100000 population whereas in women, it was of 31.26 cases per 100000 population.

**Figure 2:**
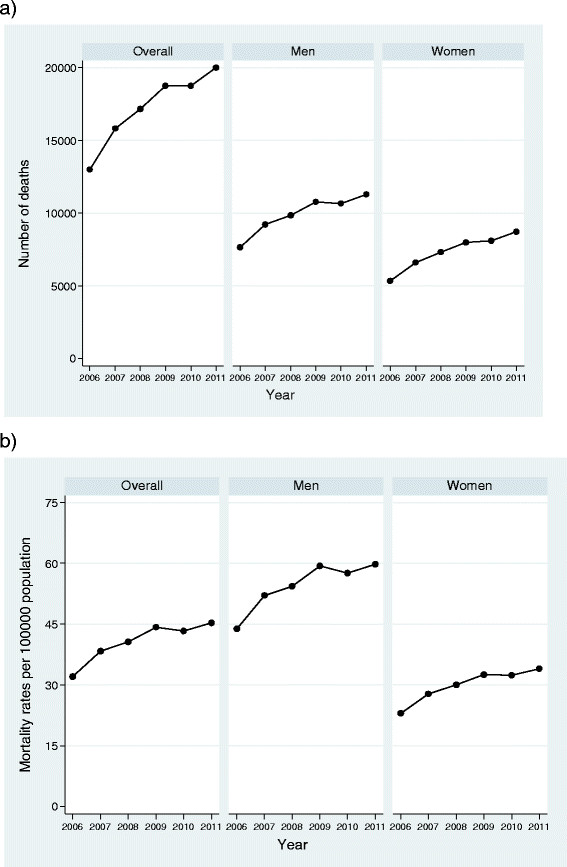
**Number of deaths and age-adjusted standardized mortality rates.** The data represent, for the entire population of severe sepsis and for men and women in a specific manner, the annual number of deaths (plot 2**a**) and the age-adjusted standardized mortality rates (plot 2**b**). Rates were calculated by direct methods based on the European standard population. For the entire population the number of deaths raised from 12988 cases in 2006 to 20012 in 2011, whereas the standardised mortality rate has gone from 32.1 cases in 2006 to 45.3 cases per 100000 population in 2011. In men, the number of deaths increased from 7650 to 11290 and the standardised mortality rate went from 43.76 cases in 2006 to 59.76 cases/100000 population in 2011. In women (5338 deaths in 2006, 8722 in 2011), the increase was higher going from 22.95 cases/100000 population in 2006 to 34 cases/100000 population in 2011 (right section).

Figure 2 shows that within these years a significant increase in standardised mortality rate has occurred, going from 32.1 cases in 2006 to 45.3 cases per 100000 population in 2011 with an APC of 6% (95% CI: 1.9, 10.3).

Figure 2 also shows the rising trend in standardised mortality rate in men going from 43.76 cases in 2006 to 59.76 cases/100000 population in 2011 with an APC of 5.4% (95% CI: 1.3, 9.6). In women, the increase was higher going from 22.95 cases/100000 population in 2006 to 34 cases/100000 population in 2011 with an APC of 6.97% (95% CI: 2.8, 11.4).

**Figure 3:**
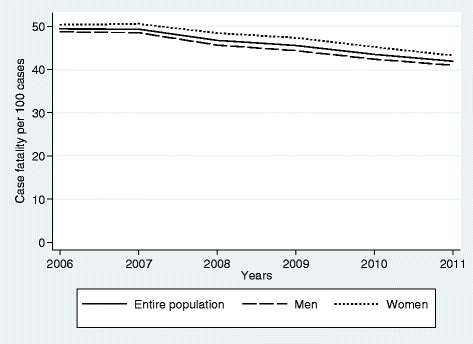
**Age-adjusted case-fatality rates of Severe Sepsis from 2006 to 2011.** Changes with time in case-fatality rate (CRF) by 100 episodes of severe sepsis. Overall rate (solid line), data in men (long-dash), data in women (short-dash). Age- and sex- standardised CFR showed a significant decrease over time going from 49.3 per 100 in 2006 to 41.9 in 2011. In men, the standardised rate has gone from 48.71 cases per 100 in 2006 to 40.97 cases/100 in 2011 whereas in women the rate has gone from 50.34 cases/100 in 2006 to 43.22 cases/100 in 2011.

Age- and sex- standardised CFR showed a significant decrease going from 49.3% in 2006 to 41.9% in 2011 with an overall APC of -3.5% (95% CI: -4.3, -0.2) (Figure 3). In men, the standardised rate has gone from 48.71 cases per 100 in 2006 to 40.97 cases/100 in 2011 with an APC of -3.7% (95% CI:-4.5, -2.9) whereas in women the rate has gone from 50.34 cases/100 in 2006 to 43.22 cases/100 in 2011 with an APC of -3.24% (95% CI: -4.2, -2.2).

## Discussion

To the best of our knowledge this is the first study to provide nationally population-based representative estimates of the epidemiological characteristics and recent trends of severe sepsis in Spain. Using information drawn from all acute-care hospitals nationwide from 2006 to 2011, this study demonstrates the growing burden of severe sepsis in Spain in recent years with a remarkable increase in its incidence and mortality rates. On the other hand, it shows a significant decreasing trend in case-fatality rates over time despite an increase in the age and severity of the affected population.

This analysis indicates that severe sepsis represents 1.1% of all hospitalizations in our country within the study period with an overall incidence rate of 87 cases per 100,000 population. Although it is well known that there is a substantial worldwide variability in the occurrence of severe sepsis [18], our figures are close to those found in related studies from the USA [5]-[7] and Australia [14]. Also, the incidence rate is in accordance with the estimation of 50-100 cases per 100,000 population recently reported for developed countries [2].

Comparison with similar Spanish studies, while local, show that our figure is greater than that observed in the Community of Valencia between 1995 and 2004 [13] but lower than those described in two studies in the Region of Madrid [12],[28]. In one of these studies, we documented a rate of 141 cases per 100,000 population in 2001 [12]. However, that study was limited to a single year and to a very specific region where large hospitals concentrate, which could explain fairly well the differences encountered. Our present work estimates the national incidence and allows us to know the extent and impact of severe sepsis nationwide overcoming the limitations of local data.

Our results also demonstrate that from 2006 to 2011 the standardised incidence rate experienced an upward trend with an annual increase of 8.6%. Though the interpretation of this increasing trend is not easy and may be in part confounded by factors such a greater awareness of severe sepsis, the introduction of specific ICM-9 codes that facilitate its coding in medical records, or other methodological issues [29], there are possible reasons for a real rise in the incidence of severe sepsis including, among others, the increased age and comorbidities of the population and the greater use of invasive procedures and immunosuppressive drugs [1],[2],[5]. Indeed, factors related to this increase remain an open question that our study is unable to resolve but our figure is rather close to that described by authors such as Martin [5] and Dombrosvky in the USA [7]. In addition, other Spanish studies, although not focused on severe sepsis, have observed a clear rise in sepsis hospitalizations in the last few years [13],[28].

As for the demographic and clinical characteristics, our results agree, in general, with international data. Thus, we find that severe sepsis is more common in men [4]-[6], who also develop it at an earlier age than women. Though the explanation for this finding remains uncertain, it is in agreement with Martin’s report [5] where men were 4-6 years younger than women. Additionally, almost two thirds of the cases are over the age of 65, with a similar percentage presenting underlying comorbidities [4],[7],[14] and regarding the number of organ failures, our results are similar to those found in international literature where about 58%-81% of cases of severe sepsis present one organ dysfunction and just 6%-16% show three or more [4],[7],[8].

Microbiological data indicate that less than a third of the cases have identified pathogens, with bacteremias coded in 16% of cases overall. Though it has to be recognized that national databases may fail to depict these data accurately, our figures fully agree with that observed in a recent study from our country [15]. In this prospective but short-term study performed in 2003 at three hospitals in Madrid only 29% (95% CI 25-32) of microbiological samples were positive, whereas a total of 12.5% of cases had positive blood cultures.

As regards to the causative organisms, Gram-negative bacteria were the most frequently involved microorganisms and there was a remarkable increase in the number of these organisms from 2006 to 2011. Though these findings could differ from the common literature that indicates that gram-positive organisms have become the most common cause of sepsis in the past 25 years [2],[5], they are in line with more recent data [1] and fully agree with those found in other studies in our country. In fact, our results confirm the microbiological profile found by Esteban et al in Madrid in 2003 [15] and the rising trend of sepsis caused by gram-negative bacteria found by Andreu et al in the Region of Valencia from 1995 to 2004 [13].

Additionally, our analysis shows that, over the study period, cases of severe sepsis occur in older people, who have more comorbidities and develop a greater number of organ failures. These changes, similar to those described in reputable USA studies [5],[7], seem to reflect the ageing and chronic morbidity of western societies [30] and paint a picture of greater frailty and severity of disease over time.

As previously reported [4]-[7],[14],[31],[32], age, comorbidities and the number of organ dysfunctions are factors significantly associated with the risk of in-hospital death in our cases. However, our fatality close to 43% is high, greater than the 21%-33% described in older studies [4],[11],[12],[14] or in local studies performed during a very short period [15]. Nevertheless, comparisons with recent studies from our country show that our rate agree with the 42.5% reported by Andreu [13] in the Region of Valencia between 1995 and 2004, and with the results of Ayala-Rodriguez [28] (40% in men, 39% in women) in the region of Madrid between 2003 and 2011. Although we realize that we cannot entirely rule out the possibility that the cases diagnosed as severe sepsis are the most critical cases for which mortality rates are higher [13],[33], nor exclude that withholding or withdrawing of treatments have occurred, our figures are visibly lower than those recently reported in the ICU setting [34],[35].

Additionally, our results show a significant decreasing trend in case-fatality rates with an average decrease of 3.5% per year from 2006 to 2011, a pattern that, in the above depicted setting of greater age and frailty of the affected population, we consider particularly important.

These data add to the most recent literature showing a decreasing trend of case-fatality in severe sepsis despite a substantial increase in the number of deaths related to the process [5]-[8], a finding that our study also confirms. As regards case-fatality rates, Dombrovsky reported annual decreases of 1.4% between the years 1993 and 2003 [6] whereas Lagu observed an average annual decline in adults of 2% from 2003 to 2007 [8]. Our rate of decline is somewhat higher, but differences in the populations analyzed and the methodology used for estimation of trends, joinpoint regression in our work, may at least partially explain the differences. We must also recognize that notable educational clinical initiatives promoting best practices in the management of severe sepsis have been developed in recent years in Spain [36], which quite possibly have been beneficial in reducing our case-fatality over time.

Lastly, a finding we consider remarkable in our analysis is the gender-related disparities in the incidence and mortality of severe sepsis. In our study, although women suffer less from severe sepsis than men, trend analysis showed a higher increase in both incidence and mortality rates from 2006 to 2011. Additionally, joinpoint analysis showed a lower decline of CFR in women than in men. Prior studies have also found similar disparities [4]-[6], but the possible impact of gender in the incidence and outcome of severe sepsis is a highly controversial topic and the reasons for these differences remain unclear [37],[38]. Although, in our study, age could play an important role, these disparities require a more in depth and detailed analysis which we are in the process of carrying out.

Among the strengths of this study we can emphasize its nationwide nature. While there is not national surveillance for severe sepsis in Spain, this study captures all acute-care, public and private, hospitalisations nationwide and serves as an adequate proxy. The data source used is considered the main information source to carry out epidemiological studies and health services research in our country as it includes data on over 97% of all annual hospital admissions [19]. To avoid errors due to changes in the ICD9-CM system we chose a period where the selected codes for severe sepsis, including the codes 995.92 and 995.91 that became effective in our country in 2004, suffered no change. Furthermore, the joinpoint regression used for analysis is a robust, widely used and validated methodology to evaluate trends. Moreover, we followed the guidelines for reporting observational studies, as outlined by the STROBE Initiative [39]. Thus, we firmly believe this study provides potentially robust data on the characteristics and impact of severe sepsis in our country. This information can be useful in the design and application of educational and therapeutic programmes to promote the early identification and treatment of patients with severe sepsis that can improve the quality of healthcare and enable a cost-effective use of health resources. At the same time, our data represent a fundamental working basis to evaluate the impact of severe sepsis in our country and estimate future needs.

These data are complementary to recent reports of trends in severe sepsis epidemiology published in North America and might be useful in other European countries with a population similar to ours.

We must also acknowledge possible limitations of our work. This study is subject to the limitations inherent in retrospective studies using administrative databases. Data from these databases lack of many measures obtainable only from chart review. Furthermore, these data do not allow causal inferences to be made. Concerning the potential risk for bias based on coding practices although we recognize that the study would be enhanced by clinical validation of diagnostic codes, access to clinical histories in our country is restricted by law [40],[41] and these may not be consulted where the aim is research, even if it is epidemiological in nature. However, the use of such databases is well-established in severe sepsis epidemiology [4]-[8],[11]-[15],[42] and has been shown to furnish valuable information for assessing the need for preventive and therapeutic care and for service planning [16]-[18],[43]. Further, the results of a recent meta-analysis clearly support the use of administrative data to monitor mortality trends in severe sepsis [43], reassuring the essential role of a consistent use of national administrative data for epidemiological monitoring of incidence and outcomes [18]. Additionally, the chief advantage of the hospital discharge database that underpins this study is that information is recorded by specialised medical coders based on a detailed review of the medical chart, increasing the likelihood of accurate documentation [44], and we must emphasize that quality assurance audits are systematically conducted on this national database to verify the adequacy of coding [19].

However, it should be recognized that although the use of ICD-9 codes has been shown to be highly sensitive for severe sepsis [18], the extracting coding strategy is not standardized and inconsistencies between various methods remain [18],[29],[33]. Different algorithms used to identify severe sepsis cases in administrative data, select cases of varying disease severity sometimes resulting in estimates, with low specificity, which tend to underrepresent the real cases of severe sepsis [29]. With this data in mind, to carry out this study we have selected and used the combination of infection and organ dysfunction codes initially described by Martin [5], and later extended by Dombrosky et al. [6],[7] after the introduction of the new ICD-9 criteria-specific codes for sepsis, severe sepsis and septic shock. The codes employed by Martin et al [5] have shown a high specificity for capturing severe sepsis cases [18],[29], with a positive predictive value of 97.7% [42]. In addition, code 995.92 has a specificity close to 100% [33]. However, as recent publications [18],[33] point out, the use of this code is limited and not recommended as a single code for the estimation of national data, but most always be combined with ICD-9 codes for infection plus organ dysfunction. Given the codes selected and the results achieved, both with respect to the incidence and mortality rates as well as case-fatality, we are confident that the cases included in our study are truly severe sepsis cases and not milder conditions.

Eventually our study may be limited by the changes in the ICD-9 system that have taken place in the last few years. Similar to what other authors believe [5],[7],[8], we should consider the possibility that these changes in the ICD-9 system may have had an influence in the data and trends observed. However, as explained above, we chose a period where the selected codes for severe sepsis suffered no change in our country. In addition, the joinpoint analysis shows a progressive increase in the respective annual percentage changes with no inflection points, suggesting that the potential changes in coding have had little effect.

Lastly, we must recognize that the restriction of the study to hospitalized patients may have introduced a bias, as Linde-Zwirble [42] points out, and our estimates of incidence rates of severe sepsis really correspond to treated incidence. In line with this, the figures of mortality associated to severe sepsis refer to hospitalized cases. A recent publication by McPherson et al. that analyses the mortality database from the National Statistics Office using the ICD-10 coding systems, points out that in England, between 2001 and 2010, 93.4% of all deaths associated to sepsis occurred within hospitals, while the remaining 6.6% took place outside hospitals [45]. We do not know whether the same occurs in our country, but it is possible that our estimates are conservative and underestimate the impact of severe sepsis in our population and our health system.

## Conclusions

This study provides, from a population perspective, the first nationally representative estimate of the characteristics and recent trends of severe sepsis in Spain. The analysis, from 2006 to 2011, of the nationwide hospital discharge database indicate the growing burden of severe sepsis in these years with a remarkable increase in its incidence and mortality rates over time. On the other hand, our data show a significant decreasing trend in case-fatality rates despite an increase in the age and severity of the affected population. There are, however, disparities in several outcomes based on both age and gender.

We believe this study provides potentially robust data on the epidemiology and impact of severe sepsis in our country. This information has significant implications for health-care service planning and may prove useful to estimate future care requirements. Additionally, it might be useful in other European countries with a population similar to ours.

## Authors’ original submitted files for images

Below are the links to the authors’ original submitted files for images. Authors’ original file for figure 1 Authors’ original file for figure 2 Authors’ original file for figure 3

## Abbreviations

APC: Annual percent change
MBDS: National minimum basic data set
ICD-9CM: International classification of diseases, 9th revision, clinical modification
ORs: Odds ratios
CFR: Case-fatality rates
COPD: Chronic obstructive pulmonary disease
