# Supplementary figures and images for: Epidemiology and recent trends of severe sepsis in Spain: a nationwide population-based analysis (2006-2011)
Source: BMC Infect Dis. 2014 Dec 21;14:3863. doi: 10.1186/s12879-014-0717-7 (PMC4327809; doi:10.1186/s12879-014-0717-7)

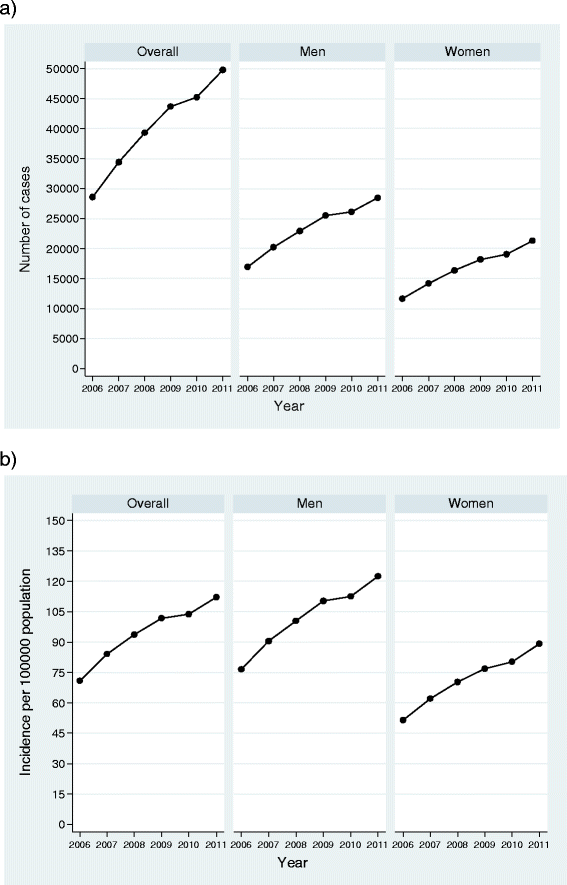

Supplement: Supplementary file 1 — Authors’ original file for figure 1 [file 12879_2014_717_MOESM1_ESM.gif]

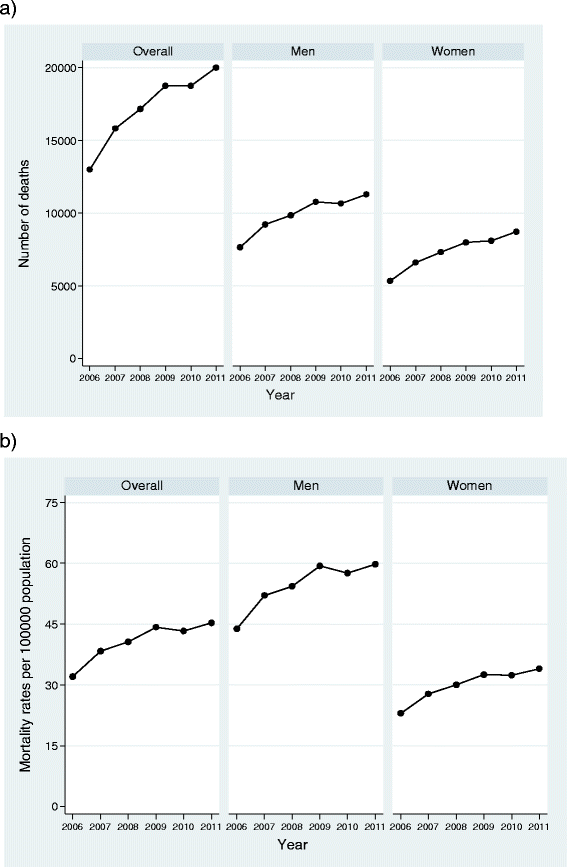

Supplement: Supplementary file 2 — Authors’ original file for figure 2 [file 12879_2014_717_MOESM2_ESM.gif]

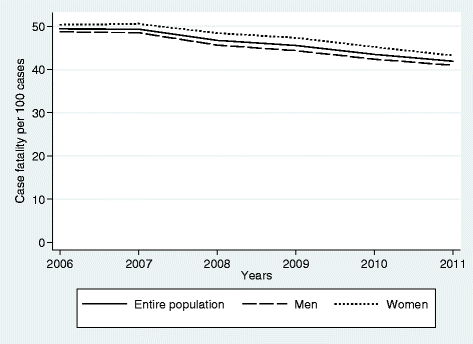

Supplement: Supplementary file 3 — Authors’ original file for figure 3 [file 12879_2014_717_MOESM3_ESM.gif]
